# Family Composition and Expressions of Family-Focused Care Needs at an Academic Memory Disorders Clinic

**DOI:** 10.1155/2013/436271

**Published:** 2013-10-22

**Authors:** Brandalyn C. Riedel, Jamie K. Ducharme, David S. Geldmacher

**Affiliations:** ^1^Department of Gerontology, Virginia Commonwealth University, Richmond, VA 23298, USA; ^2^Neuroscience Graduate Program, University of Southern California, Los Angeles, CA 90089, USA; ^3^Department of Neurology, University of Virginia, Charlottesville, VA 22908, USA; ^4^University of Alabama-Birmingham, Birmingham, AL 35294, USA

## Abstract

*Objective*. To understand who dementia patients identify as their family and how dementia affects family life. *Background*. Dementia care is often delivered in family settings, so understanding the constituency and needs of the family unit involved in care is important for determining contributors to family quality of life. *Design/Methods*. Seventy-seven families receiving care at an academic dementia clinic completed questionnaires regarding the affected person and the family. Responses were categorized as focused on an individual's needs or the family's needs. *Results*. Respondents identified a mean of 3.77 family members involved in care. Spouse (80.5%), daughter (58.4%), son (46.8%), and stepchild or child-in-law (37.7%) were the most frequently listed family members. Questions regarding the effect of dementia-related changes in cognition and mood were most likely to elicit a family-focused response. Questionnaire items that inquired about specific medical questions and strategies to improve family function were least likely to elicit a family-focused response. *Conclusions*. Both caregivers and persons with dementia frequently provided family-focused responses, supporting the construct of dementia as an illness that affects life in the family unit. This finding reinforces the potential utility of family-centered quality of life measures in assessing treatment success for people with dementia.

## 1. Introduction

Alzheimer's disease (AD) and related dementias affect an individuals' quality of life (QOL) in profound ways. QOL has been identified as a primary goal of dementia treatment [1, 2]. For instance, the International Working Group for the Harmonization of Dementia Drug Guidelines recommended that QOL be included as an outcome measure in dementia clinical trials [3]. The value of QOL measurement lies in its ability to capture potential benefits and harms of treatment not detected by typical patient-oriented performance outcomes, such as cognitive tests.

Unfortunately, the neurological deficits associated with a dementing disease often make measurement of patient QOL difficult. Anosognosia, an organically mediated unawareness of the impairments, is a frequent occurrence in the disease, affecting up to 50% of individuals with mild to moderate AD [4]. This lack of insight may limit the reliability of affected individuals' assessment of their QOL [5]. Concurrently, proxy's attributions of the affected individual's QOL are often quite different from the affected person and rated significantly lower [6, 7]. These discrepancies may help explain the lack of uniformly accepted QOL measure for studies of people with dementia. However, since both provide distinct information, a combination of proxy and patient QOL ratings may be more appropriate [8].

 Along with the importance of QOL measurement for understanding the impact of the social, behavioral, and cognitive changes associated with dementia, an accurate assignment of QOL is also important from an economic perspective. Measures of QOL serve as the basis of the “cost utility” analyses used by healthcare payer agencies to determine economic aspects of treatment effectiveness. The core of this analysis is a unit known as “Quality Adjusted Life Years” (QALYs). QALYs provide a single index that combines survival estimates and health-related QOL data, resulting in judgments about the relative effectiveness of a treatment intervention. QOL survey responses from patients were used for estimation in 58% of QALY approaches reported in one study [9]. However, QALY estimates for dementia are subject to widely different interpretations, and the utility of such estimates is controversial [10]. Valid measures of QOL in dementia will be important for assessing the efficacy of future disease modifying therapies for dementing illnesses like AD, because these agents are designed to slow progression without directly improving symptoms. Since, by definition, dementia diagnosis requires a loss of functional independence, treatments that slow progress will prolong disability. This raises significant problems for interpreting patient-centered QOL as the basis of QALY calculations for such treatments. 

 However, QOL assessment is also important from a family systems perspective. Because a family unit functions as an interconnected whole, dysfunction or illness in one family member affects other family members [11]. More than 70% of individuals with AD and other dementias receive care in a family setting; caring for someone with dementia leads to caregiver burden, adverse effects on family interactions, changes in family roles, and communication difficulties [12, 13]. The Family Caregiving Alliance has consequently suggested that quality of care assessments should embrace a family-centered perspective [14]. Therefore, development of a family quality of life (FQOL) measure is pertinent for providing a more complete basis for QALY estimates of potential disease modifying therapies for dementia, as well as for clarifying the care needs of people with dementia and their families. 

One difficulty in assessing the impact of dementia on family function and well-being lies in determining an adequate operational definition of FQOL that encompasses individual needs within a family unit. A concept primarily studied in the field of developmental and intellectual disabilities, FQOL has been defined as the “interaction and reverberation of individual members as they produce the aggregate of family quality of life” [15]. In operational terms, the Beach Center FQOL Scale [16], a measure developed to assess FQOL among families of children with developmental intellectual disabilities, conceptualizes FQOL as the positive and negative impacts “experienced by families as a result of supports and services for themselves and/or their children with disabilities.” This concept is then used to measure the effectiveness of care services [16]. 

A previous study identified that the domains of Beach Center FQOL Scale items could be successfully adapted to address dementia-related changes in family interactions [17]. The specific goals for the current study were to further explore the potential utility of the FQOL construct in guiding dementia care, and to gain insight regarding the following questions related to FQOL.

1. Who do dementia patients consider to be family?
2. Do family members report ways that dementia affects their FQOL?
3. What are the common FQOL-related needs that families identify in a healthcare setting?

## 2. Methods

### 2.1. Questionnaire Development

 Based on the Beach Center FQOL instrument and the authors' prior work with families caring for dementia, the domains associated with FQOL in dementia were defined as (1) family interactions, (2) direct care/activities of daily living support, (3) emotional/behavioral well-being, (4) physical and cognitive well-being, and (5) disability-related support/medical care [16, 18]. The domains were then used to construct a series of open-ended questions focused on assessing the impact of dementia on these areas. Developed by a neurologist (DG) and neuropsychologist (JD), this format was chosen to allow for flow of thought and feelings not traditionally captured with quantitative methods. The person with dementia and a family member were asked to complete separate but similar questionnaires. Caregivers were allowed to assist the affected person in completing the form, but were instructed to ensure that responses reflected the “affected person's thoughts.” The caregiver questionnaire form is depicted in Table 1. 

### 2.2. Data Collection

 Dyads, consisting of care recipients with dementia and family caregivers, were recruited for completion of the FQOL questionnaire during a visit to an outpatient, interdisciplinary dementia care clinic located at a university medical center. All patient participants met DSM-III diagnostic criteria for dementia, as recommended by the American Academy of Neurology guidelines for dementia diagnosis [18]. While the specific cause of dementia was not assessed for the purpose of the study, AD is the most common diagnosis among patients in the enrolling clinic, followed by a minority of patients who present with other causes of dementia such as dementia with Lewy bodies, and vascular dementia. Dementia severity, as assigned by clinicians providing care, was generally mild to moderate. Because patient responses were required, persons with severe dementia were likely not able to participate. For the purposes of this study “a family” was identified by the patient per specific guidelines to include one or more persons with whom they share emotional closeness and the dementia experience, whether or not they were related by blood or marriage. 

 The study was approved by an internal Institutional Review Board. Since the study was deemed to involve minimal risk, participants were not required to sign an informed consent document. However, before data were collected, the caregiver/patient dyads were informed of the purpose of the study and informed that each would be asked to complete a 7-item open-ended questionnaire. All participants were informed of their right to refuse participation or withdraw from the study at any time without consequence to their health care. Caregivers were invited to assist the patient in answering the questions as needed. First, the person with dementia was asked to list family members who “think of themselves as part of your family (even though they may or may not be related by blood or marriage) and who support and care for you on a regular basis.” The participants were then asked to answer the questions illustrated in Table 1. Questionnaires were completed in examination or consultation rooms that allowed for privacy. All identifying information was removed from study material before analysis to assure the participants' anonymity and confidentiality.

### 2.3. Data Analysis

Of the 81 dyads that completed questionnaires, 77 were included in the analysis. Four dyads were excluded because of insufficient data, defined as no response to >50% of questions on both caregiver and patient forms. Notably, all four of the removed dyads involved a complete lack of response from either the patient or the caregiver. Only matched pairs of patients and caregivers in which both responded to >50% of their respective question sets were used for analysis.

Qualitative methods of data analysis were used. A neuropsychologist (JD) and a graduate student in gerontology (BR) independently developed initial sets of categorization codes, which they used to separately analyze ten test cases. They then jointly reviewed their codes to determine agreement and create a final, focused, coding scheme [19]. The final scheme categorized response types into three categories (1) response focused on an individual's needs, (2) response focused on family needs, or (3) neither. SPSS for Windows version 12.0 was used to compute frequencies for types of responses and to interpret demographic information.

## 3. Results

### 3.1. Demographics

 The mean age of patients was 72.1 years, and 53% were men. For the caregiver respondents, the mean age was 62.1, and 72% were women. 

### 3.2. Defining Family

The mean number of reported family members was 3.77 (SD = 2.77). The majority of patients included their spouse (80.5%) in the listing of their family members, followed by a daughter (58.4%), a son (46.8%), a step-child or child-in-law (37.7%), and a grandchild (22.1%). Other possible family members, such as a friend, neighbor, or caregiver, were listed fewer than 10 percent of respondents answers. The frequency distribution of family member types is illustrated in Table 2.

### 3.3. Family-Focused Responses

All analyzed dyads provided at least two family-focused responses in completion of their questionnaires. This included responses that specifically mentioned multiple family members, as well as responses that included plural terms such as, “we,” “they,” or “our/my family.” Examples of family-focused responses include “We will have to repeat things. Overall, we are positive and supportive and are here to help and love our family member” and “We as a family simply repeat, re-word something he is having trouble with. Sometimes we just let it go when we know he has not “gotten” it.” Responses were considered to be individually focused when they (1) reported on changes in cognitive domains, such as memory and thinking, without mentioning the impact of those changes on others, or (2) indicated that only the patient or caregiver was being mentioned, (e.g., “Patient says she does not like being different from the way she was before the onset.” and “What can I do to improve my memory?”). Individually, only four of the patients and one caregiver who completed the questionnaires did not provide at least one family-focused response. Frequencies of family-focused responses are shown in Table 3. 

Patients were more likely than caregivers to provide family-focused responses on four of the seven questionnaire items (Items 1, 3, 4, and 5). Caregivers more frequently provided family-centered responses on two of the seven items (Items 6 and 7). One item had identical proportions of family-focused responses from both patients and caregivers.

Predictably, questionnaire Item 1, which inquired about how the presence of dementia symptoms affected family interactions, was the most likely to elicit a family-focused response, from both patients (78%) and caregivers (70%). An example response to this question from a caregiver was “My wife keeps to herself a lot. All our kids know the situation, but do not really want to accept the outcome.”

Item 5, which inquired about self-perceptions of thinking and memory, and addressed family interactions secondarily, elicited a family-focused response from 48% of patients and 32% of caregivers. An example of a caregiver's family-focused response to this question was “Poor short-term memory and repetitions limit enjoyability of discussions and family meals together. Patient has developed confabulations, in unkind ways, that disturb more distant family members.” 

Item 3 inquired about changes in mood and emotions, and the effect on family interactions. It was as likely to elicit a family-focused response from the patients as the query on cognitive abilities in Item 5 (48%) and was the second most likely question to elicit a family-focused response from caregivers (39%). Examples of family-focused responses to this Item included (From patient) “Fluctuating between normal and a little nervous. Makes kids concerned. Not as able to do things as I generally am. Feel less secure about my own input;” (From family member) it included “The limited communications are hard to deal with. The lack of initiative and empathy makes us sad, feeling like it is a one-way relationship.” 

Item 4 inquired about changes in physical functioning and its effects on family interactions. It elicited family-focused responses from 34% of patients and 23% of caregivers. An example of a patient response to this question was “Energy level has dropped, so not able to do many things. Physical part has minor effect on family interactions.” 

Items inquiring about ADL/IADL assistance (Item 2) and what questions the patients/caregivers hoped to have answered during their visit (Item 6) elicited family-focused responses in only 6–14% of patients and 14–17% of caregivers. An example of a patient family-focused response for question two was “Husband—meds. Shopping—daughter and stepdaughter. Paying bills—stepdaughter. Cooking—husband. Personal care—myself.” Examples of caregiver responses for Item 6 include “If there is any way we can communicate other than speech—sign language, and so forth.” and “Anything we can do differently that may stimulate brain more to maybe keep mind from completely going.”

Item 7 queried whether respondents saw ways for the clinic staff to help improve family function. Only 14% of patients and 26% of caregivers responded with family-focused responses that included tangible suggestions such as emotional support or increased knowledge of the disease. An example of a patient response to this question was “Better understanding of the disease process.” An example of a response from a caregiver was “Respite care—I am drowning. No help other than during the day while I am at work.” Over 60% of family-focused responses indicated family function did not need improvement.

## 4. Discussion

 The purpose of this exploratory study was to inform three key questions. The first asked about whom dementia patients consider to be family. For our respondents “family” included a mean of 3.77 members. This supports the potential usefulness of assessing FQOL in community-dwelling people with dementia instead of the more typical separate patient and single-caregiver measures of individual QOL. On many questionnaires, issues related to the dynamics of family-based care emerged (e.g., “Very difficult to communicate with my family, and this is depressing to us all”) indicating that solely inquiring about individual QOL from a caregiver or patient perspective alone fails to assess important dynamics within a family unit.

 The second study question investigated how families report the effects of dementia on domains associated with FQOL. Our earlier study revealed that family interactions were articulated infrequently in a medical care setting [17], suggesting that important aspects of family well-being may not be addressed prospectively by families seeking medically oriented dementia care or by health care providers. To compensate for this problem, the current study used questions designed to identify ways that dementia impacts family interactions in specified domains. Results indicated a high frequency of family-focused responses for items regarding thinking and memory. There were fewer family-centered responses to queries about topics to be addressed at the medical visit or on how family functioning could be improved. Persons with dementia seemed at a particular loss in expressing questions about how their family's functioning could be improved, with over half (52%) leaving the question unanswered. While it is possible that both patients and caregivers did not feel their family situation needed any improvement, there is a substantial likelihood that the observed patterns reflect a combination of ascertainment bias in our specialty clinic population, inadequate study instrumentation, and the study's physical and temporal location in an obvious medical clinic setting.

 The final question the study sought to assess was what broader needs patients and families commonly identified in a dementia-specialty healthcare setting. Though 26% of caregivers provided family-focused responses to the item querying how the clinic staff could help improve family function, few clear themes emerged from these questions. Responses were most likely to raise questions about care activities unique to their own patient and family. 

 There are study limitations that reflect larger problems in dementia care research. The majority of the study sample was Caucasian and English speaking, from a geographically restricted, mostly rural to suburban area in the Eastern US. Participants had physical and financial access to expert care and literacy levels sufficient to read and provide written responses to the questions. Our questionnaire was designed with this restricted sociocultural population in mind. Different phrasing and content for the questions would likely be needed to best address dementia care needs in other locations. These issues detract from the ability to generalize our findings to individuals from other cultures and backgrounds and point to the need for further outreach to minority and underprivileged populations regarding the effects of dementia and its associated care on family function. Additionally, the lack of randomization of study participants inherent in qualitative research further detracts from generalizability of these findings.

 Although the survey used first-person language to query the patient about their dementia care concerns, it was evident that family members frequently completed the responses survey on the patient's behalf. This was a practical necessity in the care setting because of the nature and severity of the patient's cognitive deficits. However, this limits the reliability of the data. It is also unclear as to how responses to these questions might change over time or in response to interventions. Given that dementia severity was not assessed as a variable in our study, it is possible that it would not generalize to individuals in earlier or more severe stages of dementia. Previous studies have reported that QOL may be independent of cognitive function, supporting the potential value of inquiry on aspects FQOL in all stages of the disease [20]. However, behavioral symptoms, which have a major impact on caregiver well-being and QOL, were probably not sufficiently addressed in our study. The phrasing of questionnaire Item 3, which was intended to assess these symptoms, focused on “mood,” but this may not have triggered responses regarding other behavioral and psychiatric symptoms of dementia. Future studies will be needed to assess the impact of a broader spectrum behavioral and psychiatric symptoms, especially agitation and sleep disturbances, on FQOL. 

 The use of a brief, easy to administer, open-ended questionnaire in this study was central to our examination of the elements that contribute to FQOL in dementia. This approach permitted volunteers to provide subjective views and experiences of the effect of dementia on FQOL. Future studies may be best served by asking additional domain-specific questions and inquiring about the significance of each. Additionally, measuring the frequency of family-focused responses will likely not serve as a complete indication of how dementia affects the family. Along these lines, future studies may need to assess what the family has done to cope with changes in family function that result from dementia symptoms. It is likely that many variables, such as those that reflect coping skills, social resources, caregiver and patient personality, and overall resilience, will have different effects from one family to another.

 Additional research is currently underway to further develop the FQOL construct in this population, including assessment of which FQOL domains are most important, and most affected among families caring for someone with dementia. Better understanding of these influences on FQOL in dementia has both public and personal health implications. From the public health perspective, meaningful measures of FQOL might allow refinements in “cost utility” analysis and resource utilization estimates to account for the effect of the disease on the family unit, rather than the affected person in isolation. At an individual level, assessing the determinants of FQOL may allow healthcare practitioners to be more effective in predicting the resources that families need to best support affected persons and to optimize family function and well-being. 

## Figures and Tables

**Table 1:** Study questions (worded differently for the patient versus caregiver versions).

(1) How have your memory and thinking problems affected your interactions with your family or other groups of people?	
(2) What kinds of help do family members provide with every day activities (remembering medicines, shopping, paying bills, cooking, etc.) or personal care (like bathing, eating, etc.)?	
(3) How is your mood? How have you been feeling emotionally? How does your mood affect your interactions with your family?	
(4) How are you feeling physically? How does your physical well-being affect your interactions with your family?	
(5) How is your thinking? Your memory? How have changes in your thinking/memory affected your interactions with your family?	
(6) What questions are you hoping to have us answer today regarding your care?	
(7) Are there ways that we can help improve your family's functioning as a whole?	

**Table 2:** Five most frequently reported family relationships reported by respondents with dementia.

Relationship	Frequency (%)
Spouse	80.5
Daughter	58.4
Son	46.8
Step-child or child-in-law	37.7
Grandchild	22.1

**Table 3:** Percent of family focused responses by question (*n* = 77).

Topic	Patient respondent	Caregiver respondent
(1) Effect of dementia symptoms on interactions	78	70
(2) Help with activities and personal care	14	14
(3) Mood and emotion	48	39
(4) Physical well-being	34	23
(5) Cognition	48	32
(6) Questions regarding care	6	17
(7) Improving family function	14	26
